# Motion Mitigation Techniques for Abdominal and Cardiac MR Imaging

**DOI:** 10.1002/jmri.70209

**Published:** 2025-12-28

**Authors:** Eric M. Schrauben, Gastao Lima da Cruz, Christopher W. Roy, Thomas Küstner

**Affiliations:** ^1^ Radiology and Nuclear Medicine Amsterdam UMC Amsterdam the Netherlands; ^2^ Radiology, University of Michigan Ann Arbor Michigan USA; ^3^ Lausanne University Hospital (CHUV) and University of Lausanne (UNIL) Lausanne Vaud Switzerland; ^4^ Medical Image and Data Analysis (MIDAS.Lab), Department of Diagnostic and Interventional Radiology University of Tuebingen Tuebingen Germany

**Keywords:** abdominal, cardiac, motion, motion mitigation

## Abstract

MRI of the heart and abdominal organs provides unparalleled soft tissue contrast and quantitative biomarkers, yet remains highly susceptible to physiological motion. Contractions of the myocardium, respiratory excursions, peristalsis, vascular pulsatility, and unpredictable bulk patient movement generate artifacts that impair image quality, limit reproducibility, and may necessitate repeat scans. This review summarizes motion correction strategies in cardiac and abdominal MRI, emphasizing both clinical applications and methodological principles. Techniques to address motion can be broadly categorized into prospective and retrospective approaches. Prospective methods adjust acquisition in real time, for example through respiratory or cardiac gating, navigator echoes, or external sensors, while retrospective strategies apply corrections during or after reconstruction, using k‐space binning, image registration, or model‐based reconstructions. Rigid motion, such as translations or rotations of organs, can often be corrected efficiently, whereas non‐rigid motion including myocardial contraction or peristalsis requires more sophisticated elastic registration or motion‐compensated reconstruction. Application‐specific challenges and solutions are highlighted across cardiac cine imaging, flow quantification, tagging, and quantitative mapping, as well as abdominal imaging of the liver, kidneys, and gastrointestinal tract. In each domain, examples are provided of how motion impacts diagnostic performance and how motion correction strategies can mitigate these effects. Strengths and limitations of current approaches are reviewed, from conventional breath‐holding to advanced free‐breathing motion‐resolved imaging. Emerging trends include integration of artificial intelligence with motion‐compensated reconstruction, advanced sensor technologies for real‐time tracking, and hybrid approaches combining multiple strategies. While many methods remain research‐focused, vendor‐embedded solutions and open‐source tools are increasingly available, narrowing the gap between technical advances and routine practice. Motion correction is poised to become a core feature of clinical MRI, enabling faster, more robust, and patient‐friendly examinations that reduce repeat rates, improve diagnostic confidence, and expand access to high‐quality imaging in challenging patient populations.

**Evidence Level:**

N/A.

**Technical Efficacy:**

Stage 5.

## Plain Language Summary

This review explains why motion during abdominal and cardiac MRI remains a major challenge and describes the techniques used to reduce its impact, summarizing how the heart and abdomen move during MRI. Methods to track, prevent, or correct this motion are discussed, including new advances in fast imaging and reconstruction that can improve image clarity. Better motion management can reduce repeat scans, improve diagnosis, and make MRI more comfortable for patients. This work helps guide future efforts toward more reliable, motion‐robust MRI exams.

## 1 Introduction

MRI is well‐known for its soft‐tissue contrast and the ability to quantitatively assess physiologically relevant biomarkers, such as blood flow, tissue perfusion, relaxation times (T1, T2), and diffusion characteristics. While some MRI sequences can be successfully acquired with patient cooperation, physiological motion in cardiac and abdominal MRI resulting from cardiac contraction, respiration, peristalsis, and bulk patient movement, remains a major source of image degradation. Motion artifacts have been observed in 7.5%–29.4% of scans, with repeat acquisitions required in approximately 20% of all MRI examinations, thereby prolonging exam duration, increasing costs, and contributing to non‐diagnostic studies [1]. Physiological motion can be either rigid, referring to bulk translations and rotations of the anatomy without deformation, or non‐rigid, which involves shear and elastic deformations of tissues and organs that change shape and geometry over time.

Motion artifacts manifest primarily as blurring (due to signal averaging over moving structures) and aliasing (misregistration of frequency components), including ghosting (replicas of moving objects in the phase‐encode direction). The severity of artifacts depends mostly on which k‐space samples were acquired while motion took place. In distinct and structured k‐space sampling patterns, like Cartesian, motion tends to generate strong aliasing artifacts, especially when motion occurs during the sampling of low frequencies. Conversely, sampling patterns that repeatedly sample low frequencies and/or are more incoherent, like radial or spiral, tend to generate blurring and unstructured residual aliasing or noise amplification.

To address motion‐related challenges, a range of mitigation techniques have been developed. Traditional strategies, such as patient cooperation through breath‐holding, sedation, general anesthesia, or sedation, often face limitations in routine clinical practice, especially for pediatric, elderly, or critically ill patients. Physiological triggering and gating help synchronize acquisitions with respiratory or cardiac cycles, yet residual artifacts frequently persist. This motivates the use of motion correction techniques, which are categorized broadly into prospective motion correction (PMC) and retrospective motion correction (RMC) approaches. PMC attempts to adjust data acquisition in real time to counteract motion [2], while RMC approaches apply corrections during or after image reconstruction [3].

The heart and abdominal organs move at different magnitudes and directions and with different apparent sources (Table 1). Cardiac MRI is particularly susceptible to motion artifacts from breathing, cardiac contraction and blood flow. In abdominal MRI, breathing and peristalsis introduce additional challenges per organ of interest. Both the target organ and the target application dictate how motion is dealt with. For example, for simple cardiac anatomical scanning, triggering is a good solution to “freeze” the contraction of the heart, while if cardiac‐resolved function is desired the heart needs to be “frozen” at multiple time points. However, in a field like exercise MRI where the goal is to measure the heart during stress conditions, all motion types (cardiac, respiration, and bulk movement) need to be simultaneously resolved. MRI provides a number of methods for mitigation of these different motion types, and many of them are clinically available or emerging across academic hospitals (Table 2).

**TABLE 1:** Overview of typical magnitude and sources of motion for target cardiac and abdominal organs.

Target organ	Motion source	Rigid displacement (mm)			+ non‐rigid	References
		Superior‐Inferior	Anterior‐Posterior	Left‐Right		
Cardiac	Breathing	12.1 ± 5.7	4.3 ± 3.4	2.3 ± 1.7	Yes	[4]
Liver	Breathing	10.6 ± 7.0	4.6 ± 1.6	5.2 ± 1.8	Yes	[5]
	Peristalsis	8 ± 3	3 ± 1	5 ± 2	Yes	[6, 7]
Kidneys	Breathing	11.1 ± 4.8	3.6 ± 2.1	1.7 ± 1.4	No	[5, 8]
	Peristalsis	2.4	1.2	1.2	No	[7]*
Pancreas	Breathing	9.6 ± 3.3	2.0 ± 1.2	1.6 ± 1.8	Yes	[9]
	Peristalsis	9 ± 3	5 ± 2	4 ± 2	Yes	[6, 7]
*Motility velocity (mm/s)*						
Small intestine	Peristalsis	1.8 [1.3, 2.4]				[10]*

*Note*: Values are reported as mean ± standard deviation. Breathing frequency is 10–30 breaths per minute (0.1–0.7 Hz), while peristalsis occurs at roughly 3–11 contractions per minute (0.05–0.2 Hz). *median [range] of values reported.

**TABLE 2:** Overview of motion types and clinically available mitigation methods (mostly in “Detect” and “Resolve” stage, see Figure 1) for cardiac and abdominal MRI. Emerging: Commercial products available but not widespread.

Motion type	Mitigation methods	Clinically availability
Cardiac	ECG	Widespread
	Pulse oximetry	Widespread
	Doppler ultrasound	Widespread
	Self‐gating	Research only
	(Beat) pilot tone	Emerging
Breathing	Respiratory belt/bellows	Widespread
	Lung‐liver navigator	Widespread
	In‐bore camera	Widespread
	Self‐navigation	Emerging
	Image navigator	Emerging
	Pilot‐tone	Widespread
	Millimeter‐wave radar	Emerging
	Lever‐coil motion sensor	Research only
Bulk	Center of mass	Research only
	Field cameras	Research only
	Self‐navigation	Emerging
	Real‐time	Research only
Peristaltic	Real‐time	Widespread

Abbreviations: ECG, Electrocardiogram; PPG, Peripheral pulse gating.

This review provides a comprehensive overview of motion correction techniques in cardiac and abdominal MRI. Our overview outlines motion mitigation strategies and motion characteristics, organized by motion type (Section 2), presents clinically available applications (Section 3), discusses strengths and limitations (Section 4), and highlights emerging trends and future directions (Section 5).

## 2 Overview of Techniques

**FIGURE 1:**
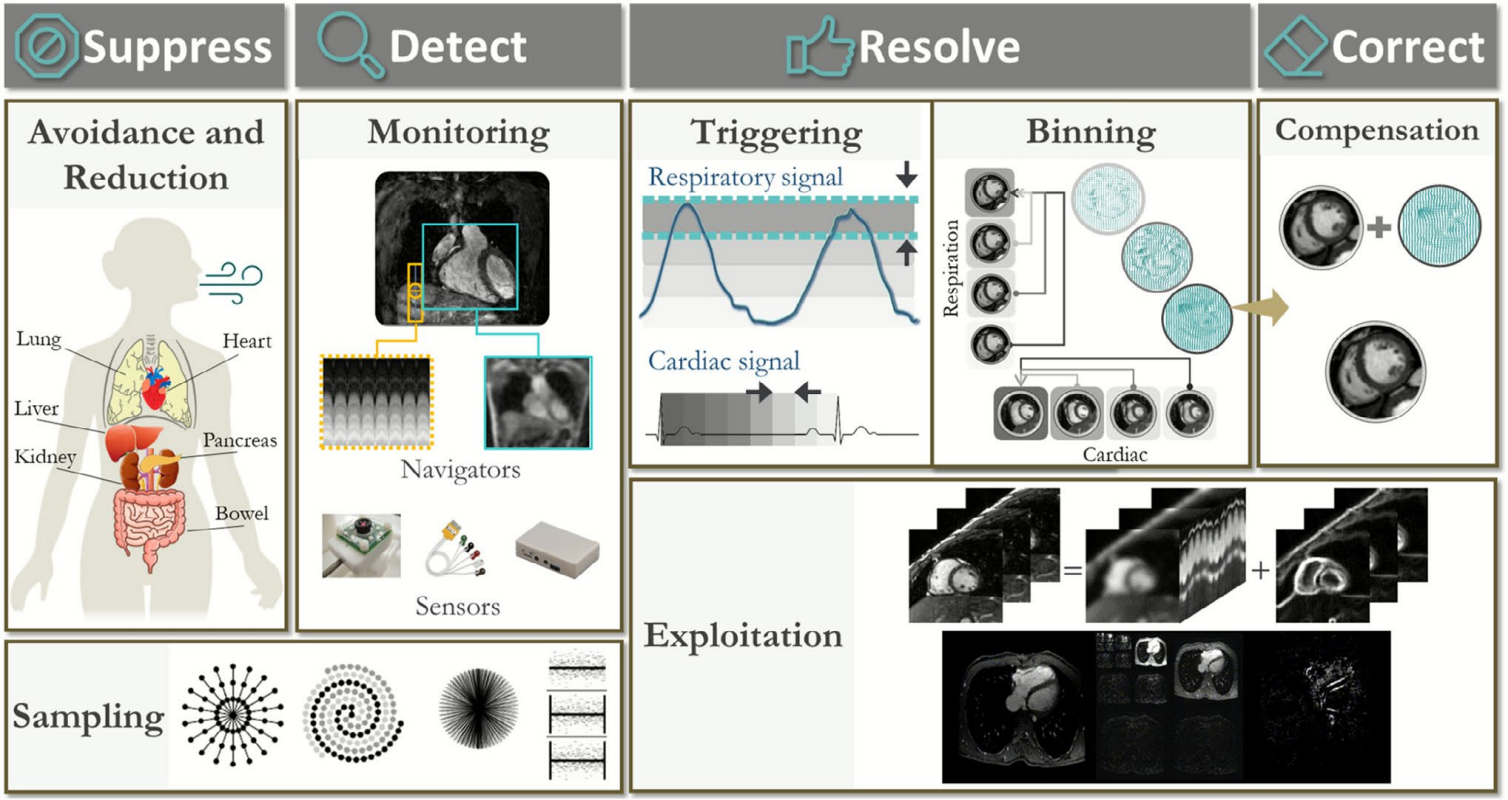
Overview of strategies to mitigate motion artifacts in cardiac and abdominal MRI, grouped into the stages “Suppress”, “Detect”, “Resolve,” and “Correct”.

When setting up and optimizing a clinical protocol, it is essential to balance spatial and temporal resolution, the imaging field of view (2D slices versus 3D volumetric coverage), scan time, and signal‐to‐noise ratio (SNR). For instance, imaging at higher spatial resolution typically comes at the cost of lower SNR or prolonged acquisition times. This trade‐off requires careful compromise to achieve clinically useful image quality within reasonable scan durations. Because physiological motion further degrades images, most imaging incorporates motion management, which can involve: (a) suppressing/avoiding motion (e.g., through breath‐holding, sedation, general anesthesia, or physical restraint) or reducing motion (e.g., via signal averaging, fast imaging, or saturation bands) [11, 12, 13, 14], (b) detecting the motion to mitigate it, (c) resolving the motion by triggering or gating acquisitions to synchronize with motion cycles, or (d) correcting or exploiting the underlying motion (Figure 1).

Fast imaging has become an increasingly important method of motion mitigation. Modern acceleration techniques, including parallel imaging (e.g., SENSE [11], GRAPPA [12]), compressed sensing [13], and more recently deep‐learning–based reconstructions [15], substantially shorten acquisition time and therefore reduce the window in which motion can occur. Highly accelerated sequences can shorten or eliminate breath‐holds entirely, reduce the number of repeated breath‐holds, and enable rapid free‐breathing cardiac and abdominal MRI that are less affected by patient compliance or fatigue.

In scenarios where patient cooperation is limited or unpredictable movements are common, incorporating one or more of these strategies into MRI protocols is crucial for maintaining image quality and diagnostic accuracy. However, each of these strategies has its limitations. For example, breath‐holding requires patient cooperation, sedation or anesthesia introduces risk and complexity, averaging increases scan time, and triggering assumes periodic motion, which may not hold for arrhythmia or irregular breathing. Computational demand for some methods can be very high, or special equipment may be needed to monitor the motion. Therefore, some methods are only emerging and/or in research state, and full clinical adoption remains an open avenue.

### 2.1 Respiratory Motion

Respiratory motion is a major source of imaging artifacts. Breath‐holding is a conventional technique to minimize respiratory motion artifacts, providing short windows of 15–20 s for motion‐free data acquisition. However, the need for repeated breath‐holds across a session [16] can lead to fatigue, misalignment of acquired slices, and poor compliance—especially in vulnerable patients [17, 18]. Moreover, breath‐holding is impractical for long acquisitions such as 3D coronary MR angiography or whole‐heart imaging, and it limits temporal resolution in dynamic studies.

Patient preparation plays an important role: clear breathing instructions and coaching enable consistent breath‐holds in cooperative individuals, while non‐pharmacological techniques like mock scanner training or feed‐and‐sleep methods can help children and anxious patients achieve motion control without the need for sedation. When cooperation is not possible, as in young children or patients with confusion, autism, or severe claustrophobia, sedation or general anesthesia may be used. Sedation is often unreliable, and anesthesia, while more effective, carries higher costs, monitoring requirements, and health risks. Consequently, these approaches are reserved for select cases, with increasing emphasis on motion‐robust acquisition strategies to reduce their necessity.

Free‐breathing methods can monitor diaphragmatic or thoracoabdominal motion and acquire data only during quiescent phases of respiration (typically end‐expiration). These respiratory‐gated or respiratory triggered approaches use navigator echoes [19], respiratory belts, in‐bore cameras [20], millimeter wave radar [21], or more recently pilot tone tracking [22] to track respiratory motion. Amplitude‐based gating accepts data within a predefined motion‐amplitude window and is therefore more tolerant of irregular breathing, but it can suffer from variable scan efficiency when the patient's baseline drifts. In contrast, phase‐based gating assigns data to specific points in the respiratory cycle, providing more consistent motion state definition and sharper reconstructions, but it is less robust with variable breathing patterns, where phase estimation becomes unreliable. While effective, these approaches suffer from low scan efficiency (~40% depending on breathing pattern), since data outside of a gating amplitude/phase are discarded and must be reacquired, making scan times longer and less predictable.

More advanced free‐breathing strategies aim for 100% scan efficiency by directly extracting motion signal from the imaging data. These utilize self‐navigation [23] or image‐based navigators [24]. Self‐navigation estimates motion directly from k‐space data, typically by continuously sampling rotating lines throughout the scan. Repeated measurements of the same k‐space points at different times provide respiratory motion information that can be used prospectively or retrospectively, though accuracy may be influenced by contributions from static structures. Image‐based navigators embedded within an acquisition offer better motion separation and correction by acquiring low‐resolution image volumes at regular intervals. Alternative strategies include optimized k‐space ordering, that is, acquiring outer k‐space during respiratory motion and central k‐space during end‐expiration [25, 26], or during reconstruction, weighting k‐space according to respiratory signal (“soft‐gating”) [27].

Once respiratory motion is estimated, motion correction algorithms handle it by a combination of one or all of the following: (a) applying translational corrections in k‐space [24, 28, 29, 30], (b) binning data into respiratory states for motion‐resolved reconstruction [31, 32, 33, 34], or (c) applying non‐rigid motion correction across states [35, 36, 37].

### 2.2 Heart Motion

Cardiac motion presents a fundamental challenge, as cyclical myocardial contraction and the movement of great vessels introduce dynamic changes throughout the cardiac cycle. This motion also contains inherent diagnostic information (such as left ventricular contractility and blood flow rates), creating strong motivation to resolve it through synchronization between image acquisition and the cardiac cycle.

Pharmacological agents are rarely used for motion management, though stress‐inducing drugs are applied in cardiac stress testing and beta‐blockers may be given for coronary MRA. Patient preparation focuses on electrocardiogram (ECG) lead placement and physiological monitoring, yet ECG synchronization can fail in arrhythmias or under electromagnetic interference. Pulse oximetry‐based gating offers a simpler and more robust alternative but suffers from delayed waveform timing and reduced accuracy in patients with poor perfusion. An emerging option is MR‐compatible Doppler ultrasound, which can be used for both prospective and retrospective gating and may overcome limitations of ECG‐based methods, although its use outside fetal cardiac imaging remains limited [38].

**FIGURE 2:**
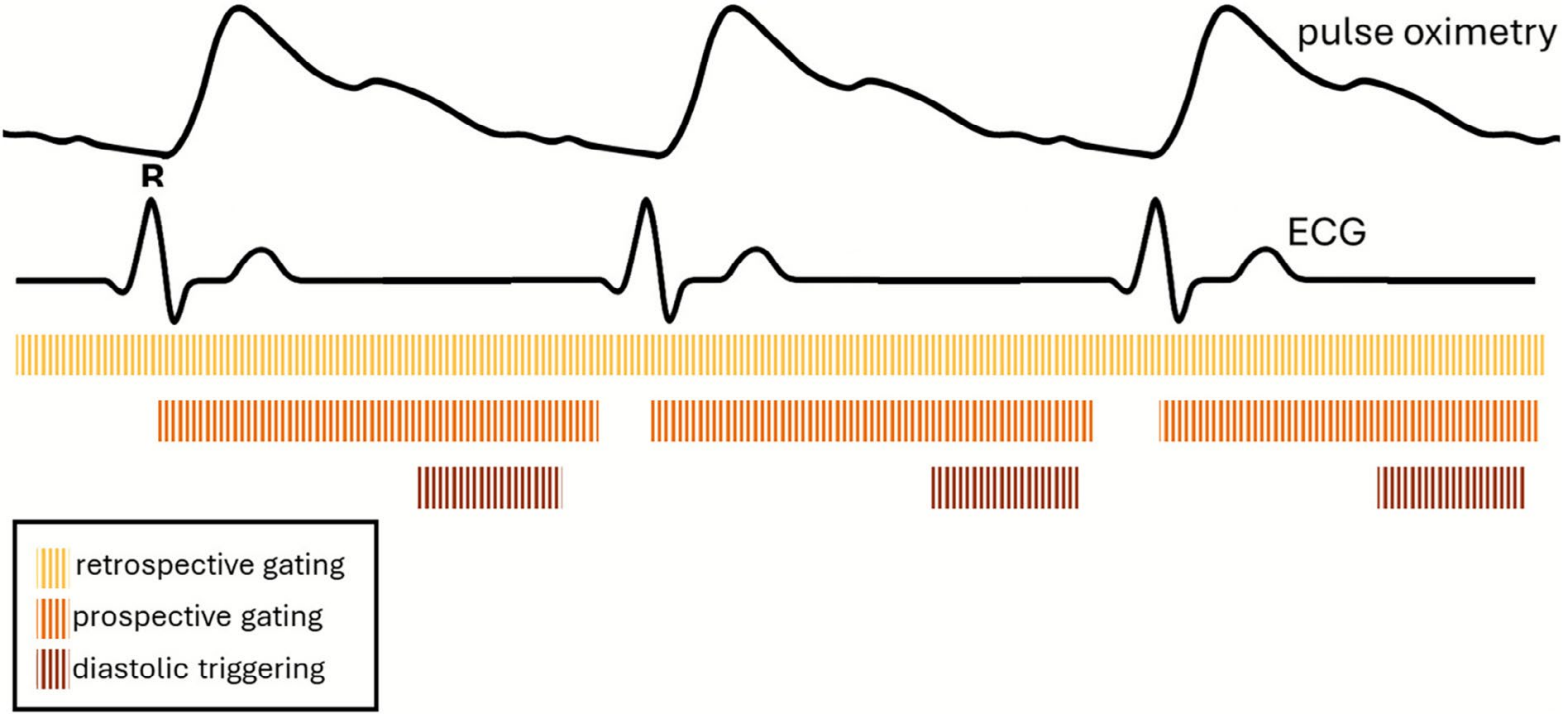
Example detected cardiac motion signals using clinically available hardware and techniques for gating or triggering MR acquisitions to these signals. ECG, Electrocardiogram.

Most cardiac MR acquisitions are synchronized using ECG signals. Prospective gating and triggering (to e.g., one diastolic phase) initiates data acquisition after detection of the R‐wave to capture specific cardiac phases [39], while retrospective gating continuously acquires data and retrospectively assigns it to cardiac phases using the ECG trace [40, 41] (Figure 2). Prospective methods offer efficiency and exact timing but can miss irregular beats and fail if the timing is unpredictable, while retrospective methods provide flexibility under irregular physiological motion at the cost of less exact timing. Prospective gating methods can increase scan time significantly by pausing during unfavorable motion phases, whereas retrospective gating acquires data continuously and thus often shortens the effective scan time.

Emerging self‐gating methods attempt to extract cardiac motion information directly from the imaging data [31, 42]. Additionally, contactless external sensors such as pilot tone have been explored for robust cardiac motion tracking [43]. Motion correction algorithms in this domain thus rely on the appropriate synchronization of the data to align with specific cardiac phases, either prospectively or retrospectively.

### 2.3 Combined Cardiac and Respiratory Motion

Combined cardiac and respiratory motion is particularly problematic in thoracic and abdominal imaging, where both processes interact. Pharmacological interventions are not typically employed for this combined motion suppression. Patient preparation remains minimal, since free‐breathing and free‐beating acquisitions are used.

“Free‐running” techniques have been developed to eliminate the need for both ECG and respiratory synchronization [31, 32, 44]. These approaches continuously acquire data during free‐breathing and free‐beating conditions, with motion signals estimated retrospectively from self‐navigation, pilot tone, or external sensors. Data are then sorted (binned) into respiratory and cardiac phases, forming a multidimensional dataset for image reconstruction. Respiratory phase binning may be followed by direct translational [24, 28, 29, 30] or non‐rigid [45] motion correction in k‐space, or used to inform non‐rigid motion correction at the image level [46]. The final reconstruction exploits spatiotemporal correlations using motion‐regularized (i.e., constraining the temporal motion direction with e.g., finite differences) [33], motion‐compensated (i.e., co‐registering using motion fields extracted with image registration between phases) [47, 48], or explores low‐rankness [49, 50] of the dynamic data (i.e., compressing the temporal motion direction into a lower dimensional representation) to generate high‐quality images.

While gating is effective under regular motion conditions, it relies on periodicity and fails with arrhythmia or irregular respiration [51], or when motion dynamics must be captured such as during interventional or physiological stress MRI. In such situations, real‐time CMR offers an alternative: fast 2D acquisitions such as spoiled gradient recalled echo (spGRE) or balanced steady‐state free precession (bSSFP) allow achieving sub‐second temporal resolution and are more robust to motion [52]. Advanced sampling and reconstruction techniques can further improve image quality, though often at the expense of spatial coverage, resolution, or SNR.

### 2.4 Bulk Motion: Non‐Periodic Patient and Organ Displacement

Bulk motion, including non‐periodic large‐scale movements such as patient repositioning, involuntary shifts due to discomfort, coughing, and swallowing, poses significant challenges in MRI. Bulk motion is particularly challenging during lengthy acquisitions or in populations unable to remain still (e.g., pediatric or critically ill patients). Unlike rhythmic cardiac or respiratory motion, bulk motion is unpredictable and can lead to substantial image artifacts if unaddressed. This often results in the need for repeating acquisitions, which prolongs scan time, reduces efficiency, and may still not lead to adequate quality if the patient cannot comply.

Patient preparation, including careful positioning, immobilization, and reassurance, can help reduce gross movements, but compliance in pediatric or critically ill patients often remains limited. As discussed above (2.1 Respiratory Motion), in such cases, sedation or general anesthesia may be considered, though these approaches are reserved for situations where other strategies are not feasible.

Sequence optimization may include fast or segmented acquisitions to limit the impact of sporadic movement, though effectiveness is constrained when motion is abrupt.

PMC methods include real‐time motion tracking using MR navigators or external sensors that allow for immediate adjustment of imaging parameters to accommodate patient movement [53]. A variety of RMC approaches exist to perform post‐processing corrections after data acquisition. Techniques such as image registration align misaligned images by estimating and compensating for motion, effectively suppressing blurring artifacts and misregistration. Additionally, advanced algorithms utilizing entropy‐based metrics can automatically correct in‐plane bulk motion artifacts by optimizing image sharpness and consistency [54].

### 2.5 Peristaltic Motion

Peristaltic motion, characterized by involuntary contractions of the gastrointestinal tract and the ureter, presents a significant challenge in abdominal and pelvic MRI. Unlike cardiac or respiratory motion, peristalsis is inherently non‐periodic and unpredictable, leading to artifacts such as blurring and ghosting, which can compromise image quality and diagnostic accuracy [2].

Pharmacological intervention plays a central role in managing peristaltic motion. The administration of anti‐peristaltic agents, such as hyoscine butylbromide (Buscopan), has been shown to effectively reduce bowel motion during MRI [55]. Intravenous administration of Buscopan results in small bowel paralysis within approximately 20 s, effectively suppressing peristaltic motion during imaging. Similarly, motion artifacts from ureter peristalsis can be mitigated through the use of a spasmolytic agent. However, readers found mixed findings, noting sharper anatomy with anti‐peristaltic use, while others saw minimal difference. Glucagon can provide a 10–20 min imaging window where duodenal motion is suppressed. Patient preparation includes fasting prior to MRI, which can reduce the frequency and intensity of peristaltic contractions. For example, fasting for 4–6 h before the scan is commonly recommended to minimize bowel activity and improve image quality [56]. However, this approach may not be suitable for all patients and should be considered on a case‐by‐case basis.

Sequence optimization emphasizes fast imaging sequences, such as single‐shot echo‐planar imaging (EPI) [57] or turbo spin‐echo (TSE) sequences [58], that minimize the impact of peristaltic motion by reducing acquisition time. Additionally, techniques like non‐Cartesian k‐space sampling are less sensitive to motion artifacts and can be beneficial in imaging regions affected by peristalsis [59].

Motion correction algorithms can be applied retrospectively through image registration [60], aligning images acquired at different time points, or motion‐state reconstruction for motion captured in a controlled way [59]. Beyond binned motion‐state images, real‐time imaging allows modeling of peristaltic motion for better understanding and mitigating its effects [61].

## 3 Applications

### 3.1 Cardiac

#### 3.1.1 Cine, Flow

**FIGURE 3:**
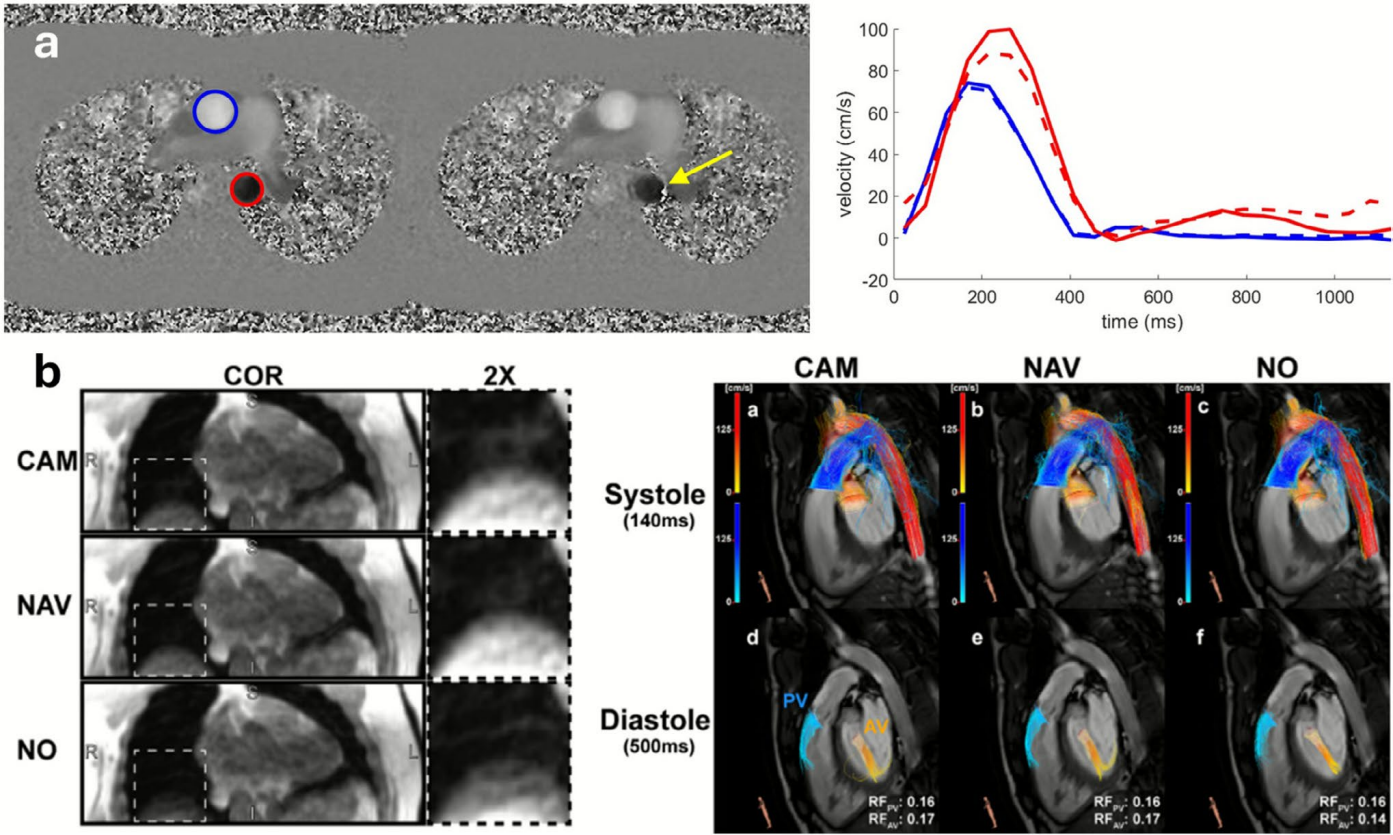
Motion in cardiac flow MRI. (a) Effects of misgating on 2D phase contrast MRI. Velocity images at peak systole for correct gating (left) and misgating (middle), in which 5 of the 16 heartbeats during acquisition were corrupted by arrhythmia, causing additional noise in the descending aorta (yellow arrow). The effects of this misgating (right) are substantial in the descending aorta (red), leading to incorrect velocity measurements (dashed line). (b) Example 4D flow magnitude coronal images using in‐bore camera‐based (CAM) gating, navigator‐based (NAV) gating, and no respiratory gating (NO). The liver‐lung interface (dotted box) shows improved delineation in both respiratory gating techniques. Systolic and diastolic streamlines (right) of CAM, NAV, and NO data sets. Slightly higher regurgitation fraction (RF) through the aortic valve (AV) was found after respiratory gating. PV: Pulmonary valve. Adapted from [20].

Cardiac cine MRI, employing bSSFP contrast, is fundamental for assessing global and regional ventricular function, wall motion abnormalities, and chamber morphology. Additionally, phase‐contrast (PC) MRI, particularly 2D and 4D flow techniques, enables quantitative assessment of flow patterns, valve regurgitation, and shunt volumes. These applications are critically sensitive to cardiac motion which must be effectively synchronized or compensated to avoid phase dispersion, spatial blurring, and underestimation of flow velocities (Figure 3a). ECG gating is the standard for temporal alignment across cardiac cycles. Typically, retrospective gating is favored in 2D cine, 2D flow, and 4D flow MRI to accommodate variable heart rates and permit reconstruction across the full cycle [62].

Short scans (2D cine, flow) are performed in a single breath‐hold [63], while for longer sequences (whole‐heart cine, coronary MRA, and 4D flow MRI), monitoring respiratory positioning and prospectively or retrospectively gating to end‐expiration is employed (Figure 3b) [20].

#### 3.1.2 Tagging

**FIGURE 4:**
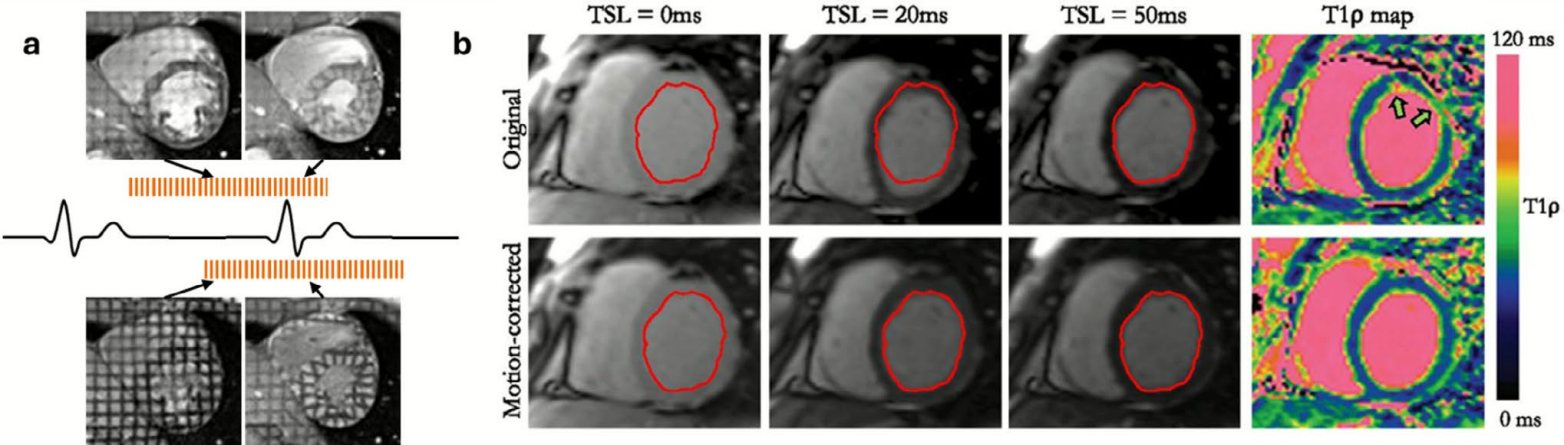
(a) Effect of tagging timing; tagging relies on consistent cardiac phase alignment. When tagging lines are set too early in diastole (top), due to, for example, poor ECG gating/timing, tag lines may degrade before reaching end‐systole and result in compromised strain analysis. Correct timing (bottom) results in clear, persistent tag lines into systole. (b) Failed breath‐holding (top) over a 13 s acquisition results in misregistered spin lock timing (TSL, showing 3 of 5 TSL points) myocardial positions and noisy T1ρ maps at the free‐wall (arrows). With non‐rigid registration of TSL points (bottom), T1ρ maps are visibly improved. Adapted from [65].

In cardiac magnetic resonance tagging, a high contrast grid‐like nulled signal pattern is used to visualize myocardial motion and measure myocardial deformation parameters such as strain. Since these “tags” recover signal according to myocardial T1, the effect fades over time, requiring re‐tagging and limiting temporal resolution of the technique [64]. Cardiac motion itself is the signal of interest in this context, but inaccurate gating or poor temporal resolution can result in tag fading, particularly if tags were not initially fully nulled (Figure 4a). This can result in spatial misregistration and loss of strain information. Single slice tagging is performed within a breath‐hold to eliminate respiratory motion [63].

#### 3.1.3 Quantitative Mapping

Quantitative parametric mapping of myocardial tissue properties (e.g., T1, T1ρ, T2, T2, extracellular volume [ECV]) offers insights into edema, fibrosis, and iron overload. These techniques require precise pixel‐wise temporal registration across multiple inversion times (T1), echo times (T2/T2), or spin‐lock times (T1ρ). Even small‐scale motion, due to inconsistent cardiac gating, can introduce partial volume effects and fitting errors, which severely affect quantitative accuracy.

Prospective motion control is typically achieved through ECG‐triggered, breath‐held imaging. For T1 and T2 mapping, single‐shot acquisitions are acquired at fixed cardiac times (late‐diastole) across consecutive heartbeats. However, mis‐triggering, failed breath‐holding, or change in breath‐hold position can induce registration errors between source images which can be corrected through in‐plane non‐rigid registration [63, 65] (Figure 4b). Gated acquisitions or motion‐compensated reconstructions are therefore essential to preserve spatial fidelity and minimize fitting errors. For whole‐heart applications, rigid translational correction of respiratory motion has been shown [29]. Finally, as ECV requires both pre‐/post contrast T1 data, images need to be motion corrected and then co‐registered.

#### 3.1.4 Late Gadolinium Enhancement

Late gadolinium enhancement (LGE) imaging is performed several minutes after intravenous gadolinium administration to visualize myocardial damage, as contrast clears rapidly from healthy myocardium but persists in diseased tissue with expanded extracellular space. LGE images are typically acquired using inversion recovery sequences, and precise timing to null the myocardium is performed with an inversion time scout. Images are collected in standard cine planes at end‐diastole, usually requiring breath‐holds of ~10 s per slice and a total scan time of 5–10 min [16, 63].

#### 3.1.5 Quantitative Perfusion

Myocardial perfusion imaging is central to the evaluation of patients with known or suspected coronary artery disease and other conditions such as anomalous coronary artery origins and Kawasaki disease. Because this technique requires continuous imaging to capture the arterial input function (AIF) and contrast kinetics, respiratory motion is inevitable and must be addressed to ensure reliable data quality. Recommendations point to encouraging free shallow breathing during acquisition [66]. To ensure complete first‐pass coverage, a sufficient number of time frames must be acquired, ideally sampling every heartbeat. Similar to quantitative mapping, non‐rigid image registration should be used to align both AIF and myocardial images across time frames. This correction should also extend to proton density–weighted images, which serve as a reference for normalization.

### 3.2 Liver

Liver MRI is performed for focal lesion detection/characterization, chronic liver disease staging, transplant assessment and treatment planning. Motion in the liver is predominantly caused by respiration, with secondary impacts from cardiac pulsation (especially in the left lobe) and bulk motion. Anatomical liver imaging to assess lesions is performed using T2‐weighted, T1‐weighted, and DIXON images during breath‐hold, while respiratory navigator triggering can be used for intermediate T2‐weighted turbo spin‐echo sequences [67].

**FIGURE 5:**
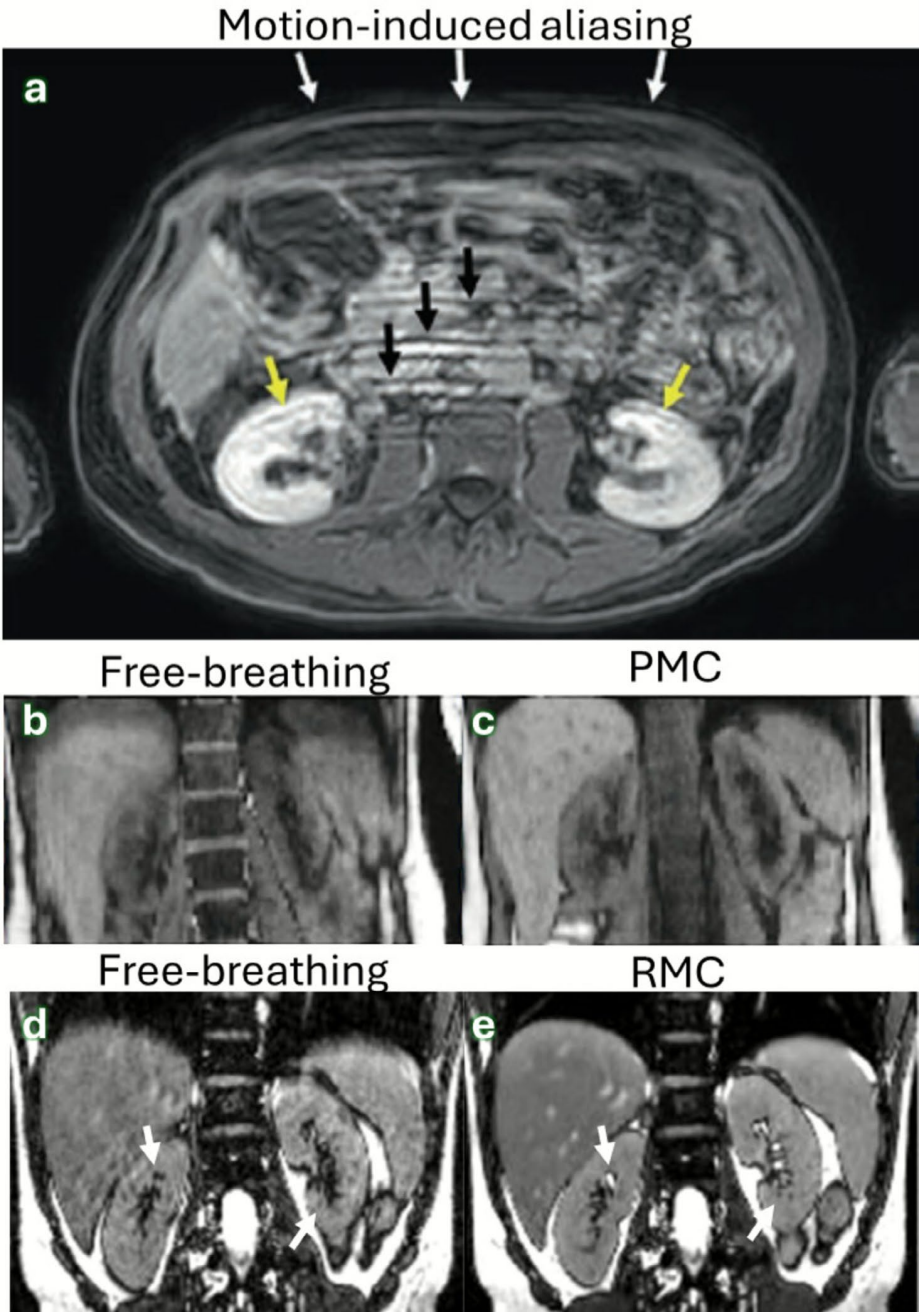
Breathing motion in liver and kidney MRI. (a) A failed breath‐hold in a Cartesian T1‐weighted Dixon GRE results in replicating the fat boundaries throughout the FOV (ghosting). (b) Free‐breathing 3D stack‐of‐stars T1‐weighted GRE scan demonstrates blurring, concealing liver and kidney boundaries. (c) Data from (b) prospectively motion corrected (PMC) using free‐induction decay echoes. (d) Blurring from radial imaging as in (b), now from a T2‐weighted acquisition. (e) Data from (d) retrospectively motion corrected (RMC). This approach incorporates all of the data into the reconstruction of a single image, achieving higher SNR. Adapted from [68].

Abdominal subcutaneous fat is a common source of motion artifacts since this tissue moves substantially during respiration and has high signal in most sequences (Figure 5a). This moving fat generates strong coherent aliasing artifacts that propagate along the phase encoding direction. Breath‐holding remains the first line of defense against respiratory motion, despite limiting scan times to < 20 s. While this is feasible for many 2D sequences, it limits spatial resolution and coverage in 3D sequences. Therefore, respiratory triggering/gating is commonly used [69]. The main downside of these approaches is the low scan efficiencies < 50%, which has prompted research efforts to develop motion robust, efficient solutions.

(Semi‐) Quantitative liver MRI includes diffusion‐weighted imaging (DWI), intravoxel incoherent imaging (IVIM), MR elastography (MRE), and dynamic contrast enhanced (DCE) imaging. In DWI, IVIM, and MRE, single‐shot EPI is used to acquire during a breath‐hold (which limits *b*‐value/wave offset sampling and SNR), with free‐breathing (more motion artifacts), or with respiratory‐triggering (resulting in longer scan time) [67, 70]. Cardiac motion can degrade left‐lobe DWI and confound ADC estimation, motivating careful gating/trigger selection. To track contrast dynamics over several minutes of inflow into the liver, free‐breathing approaches are preferred, though breath‐holding may be performed for non‐quantitative DCE [67].

**FIGURE 6:**
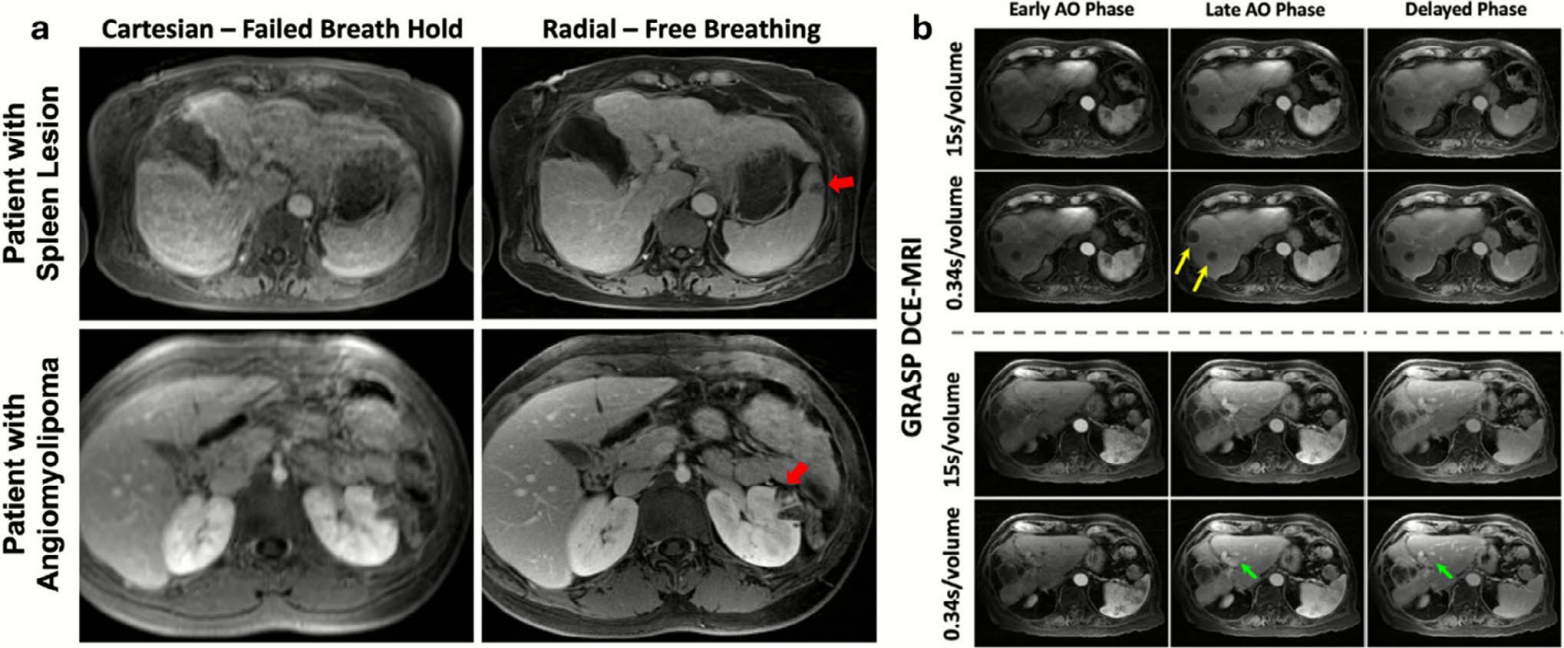
Benefits of radial imaging in liver imaging. (a) While a failed breath‐hold in Cartesian image is highly sensitive to motion, free‐breathing approaches coupled with radial sampling can produce high image quality for assessment of lesions. (b) Advanced reconstruction of radial data using model‐based assumptions about contrast characteristics can resolve contrast dynamics over a 3D volume at high temporal dynamics. Adapted from [71].

PMC strategies include ECG when dynamic timing relative to the cardiac cycle is critical and respiratory synchronization to a consistent breathing phase; several liver perfusion protocols begin with breath‐hold dynamics to reduce early motion and then continue in free‐breathing [70]. Non‐Cartesian imaging has become a clinically established free‐breathing approach for liver DCE and T2‐weighted imaging but still suffers from respiratory‐induced blurring and incoherent artifacts (Figure 5b). Nonetheless, these frameworks benefit from the inherent motion insensitivity of radial sampling, and in the case of GRASP, continuously acquire data which can be flexibly reconstructed at variable temporal resolutions (Figure 6) [71].

RMC approaches remain essential when fully motion‐free acquisition is impractical. In liver studies, image registration is routinely integrated into quantitative pipelines (e.g., for function mapping with hepatocyte‐specific agents or for whole‐liver analyses), aligning free‐breathing series before parameter estimation to curb motion‐induced bias [72]. For dynamic contrast studies and other time‐series, data can be temporally sorted and co‐registered across frames to stabilize pharmacokinetic modeling under free‐breathing conditions [70].

### 3.3 Kidney

Kidney MRI is commonly used for indeterminate renal masses, chronic kidney disease, kidney transplants, and pre‐operative planning. Kidney MRI is sensitive to cardiac/pulsatile motion and some bowel/ureter peristaltic motion, but especially to respiratory and bulk motion. The respiratory‐induced displacements of the left and right kidney are about ~65% and ~70% of the liver displacement (respectively) and are typically under 10 mm [5].

**FIGURE 7:**
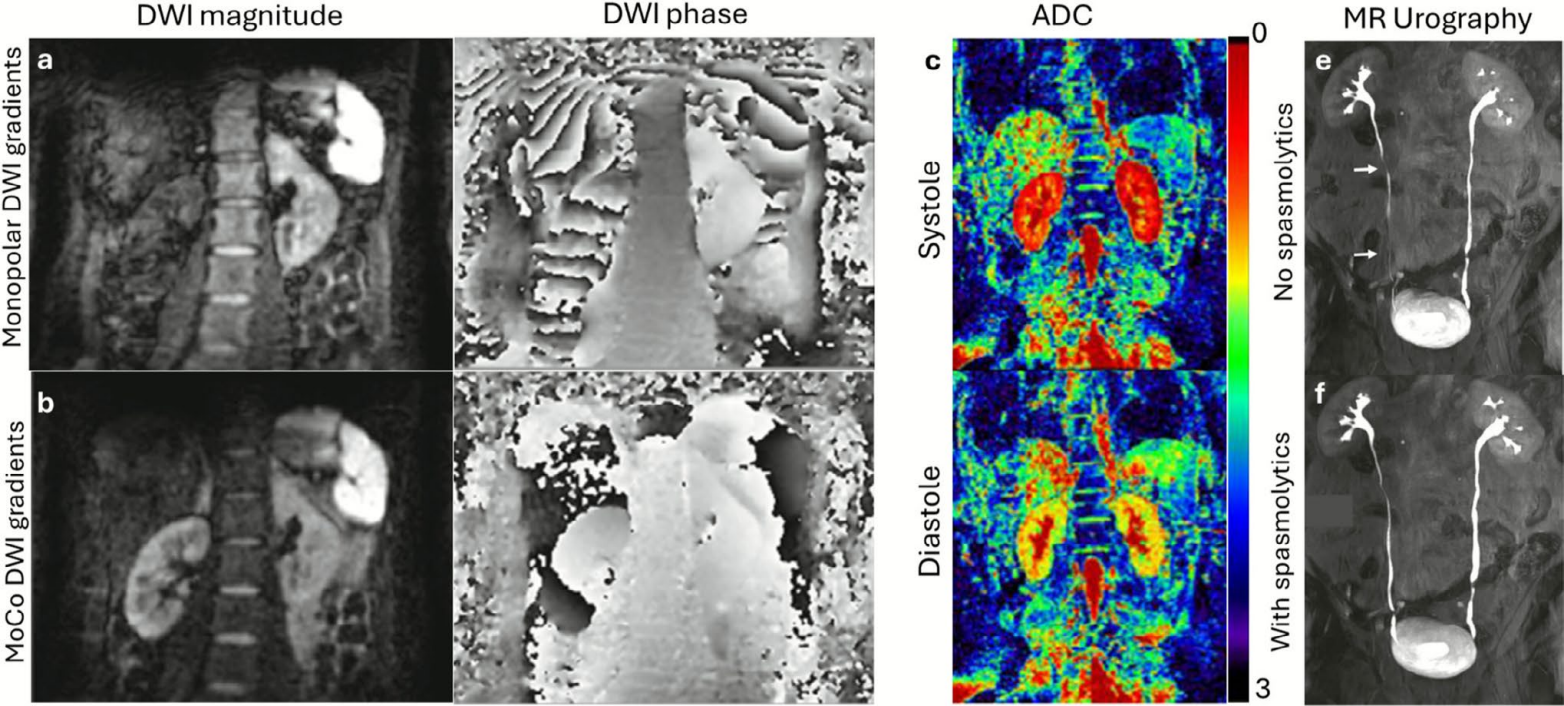
Respiratory, cardiac, and peristaltic motion effects in kidney MRI. (a and b) Renal examples of signal void and phase variability induced by motion in diffusion weighted imaging (DWI), along with a prospective approach using velocity compensated diffusion gradients that reduces these effects. (c and d) As the kidneys are highly perfused, apparent diffusion coefficient (ADC) maps display cardiac pulsation‐induced variability. (e and f) Motion artifacts from ureter peristalsis can be mitigated through the use of spasmolytics. Adapted from [68, 76, 77].

Similar to liver MRI, PMC in the kidney has been achieved using navigator echoes, a respiratory belt, and more recently with free‐induction decay navigators (Figure 5b,c) [73]. PMC has also been achieved using built‐in diaphragmatic navigators [74] and self‐navigated radial trajectories like PROPELLER [23] and BLADE [75]. Some MR applications like DWI are particularly sensitive to both respiratory and cardiac motion, where the motion can create signal voids, phase inconsistencies, and changes in contrast (Figure 7a,b) [76]. The velocity of the motion causes artifacts that can be ameliorated through motion‐compensated diffusion gradients to minimize these effects [78].

B0 inhomogeneities also change during respiration, which can induce breathing‐specific geometric distortions. These challenges have also been tackled via diaphragmatic navigators to derive respiratory‐resolved B0 maps to correct distortions [79].

As in the liver, the kidney is a highly perfused organ in which cardiac and pulsatile motion remains as secondary sources of artifacts; ADC measurements have been shown to vary during the cardiac cycle (Figure 7c,d) [77, 80]. Cardiac induced pulsatile motion has been leveraged into diagnostic information via IVIM or ASL [74]. Peristalsis can also impact certain kidney MRI protocols. While the kidneys are partially shielded from bowel motion, they remain susceptible to prolonged ureteral peristalsis, which may last several minutes. In such cases, spasmolytic medication can help reduce blurring artifacts that would otherwise obscure ureter visualization (Figure 7e,f).

RMC can also be used in kidney MRI in the form of respiratory‐resolved imaging, in which data is grouped into different respiratory phases using reconstructions that leverage temporal redundancy, for example, XD‐GRASP [33]. Such approaches have been applied to enable free‐breathing DCE where high temporal resolution is required [81]. For quantitative DCE, on the other hand, a co‐registered series is desired to ensure a reliable fit to the pharmacokinetic model. Multi‐contrast registration can be deployed for this task, enabling quantitative kidney DCE from free‐breathing data [82]. Respiratory corrected reconstruction is another type of RMC where the patient's motion is incorporated into the reconstruction process, removing motion artifacts in the process (Figure 5d,e) [47]. In the context of kidney imaging, such approaches have been applied to anatomical [83] and DCE imaging [84]. Notably, RMC enables fully elastic motion correction, whereas PMC is limited to affine models.

### 3.4 Pancreas and Hepatobiliary System

**FIGURE 8:**
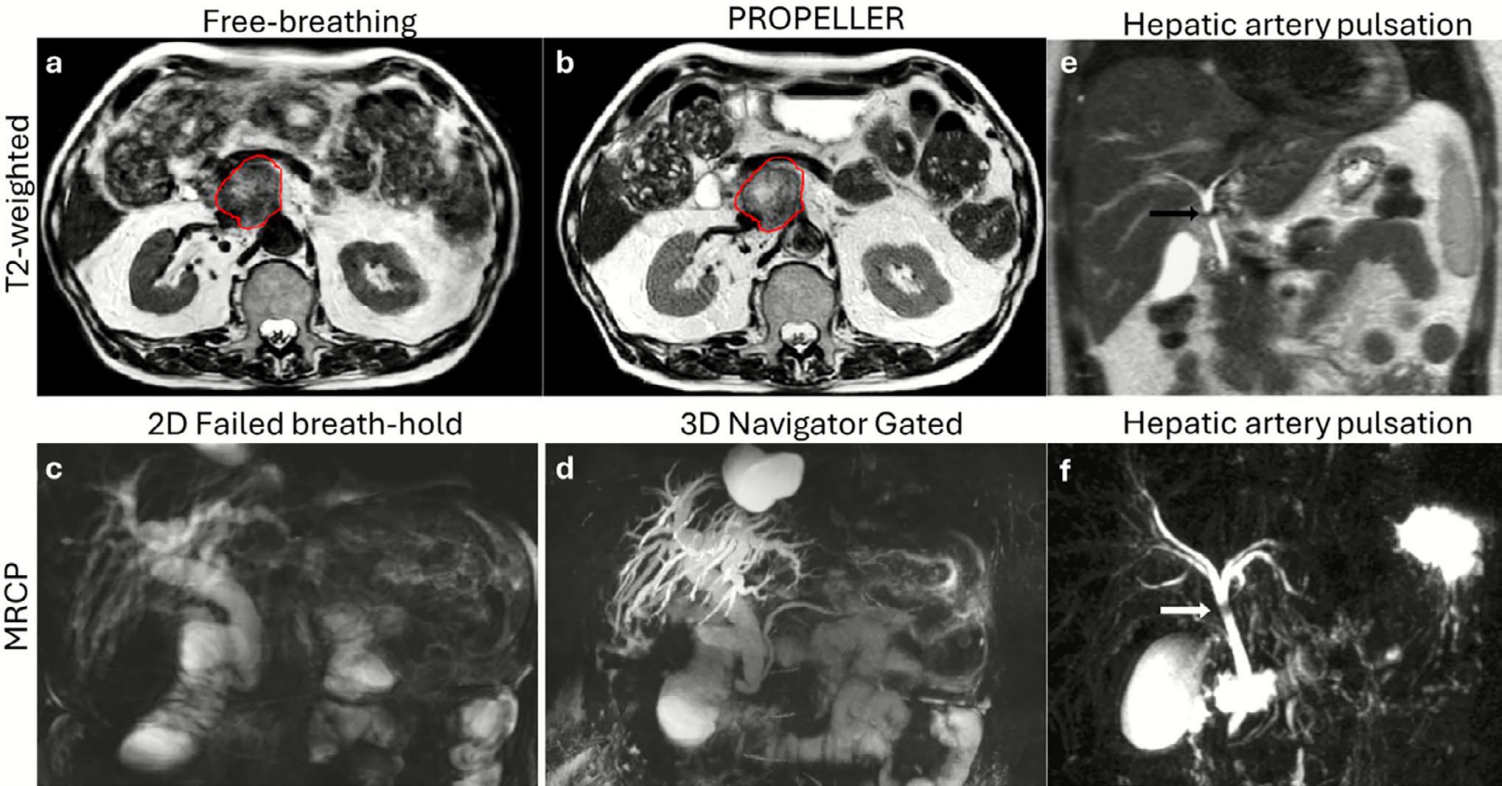
Pancreas and MRCP applications of motion mitigation. (a and b) Free‐breathing T2‐weighted imaging of a pancreatic tumor (red contour) shows blurring and residual aliasing, which is improved through a PROPELLER (radial blades) acquisition. (c) MRCP in multiple 2D slices in which several slices exhibit respiratory failure, causing apparent discontinuities and stenosis of the ducts mimicking pathology. (d) Navigator gated 3D MRCP can produce high‐quality images of the hepatobiliary tree provided the patient's breathing pattern is consistent. (e and f) Example pulsation artifacts causing a signal void on MRCP, creating an apparent narrowing of the common hepatic duct. Adapted from [86, 87].

MR is a useful modality to image the pancreas, gallbladder, bile ducts and surrounding hepatobiliary system, where it can assess pancreatitis, non‐alcoholic fatty pancreas, pancreatic cancer, gallstones, and the pancreatic/biliary ducts [85]. The pancreatic and hepatobiliary trees are commonly assessed with MR Cholangiopancreatography (MRCP), a dedicated protocol focused on heavy T2‐weighted HASTE/TSE (i.e., TE > 600 ms and TR > 3000 ms), supported by T1 Dixon, DCE and in some cases MRE. MRCP is particularly sensitive to respiratory and bulk motion, although some sequences are also sensitive to cardiac/pulsatile motion and bowel peristalsis. The pancreas deforms and moves significantly [6, 7, 9], which can lead to significant blurring and be mitigated through PROPELLER type acquisitions [86] (Figure 8a,b).

**FIGURE 9:**
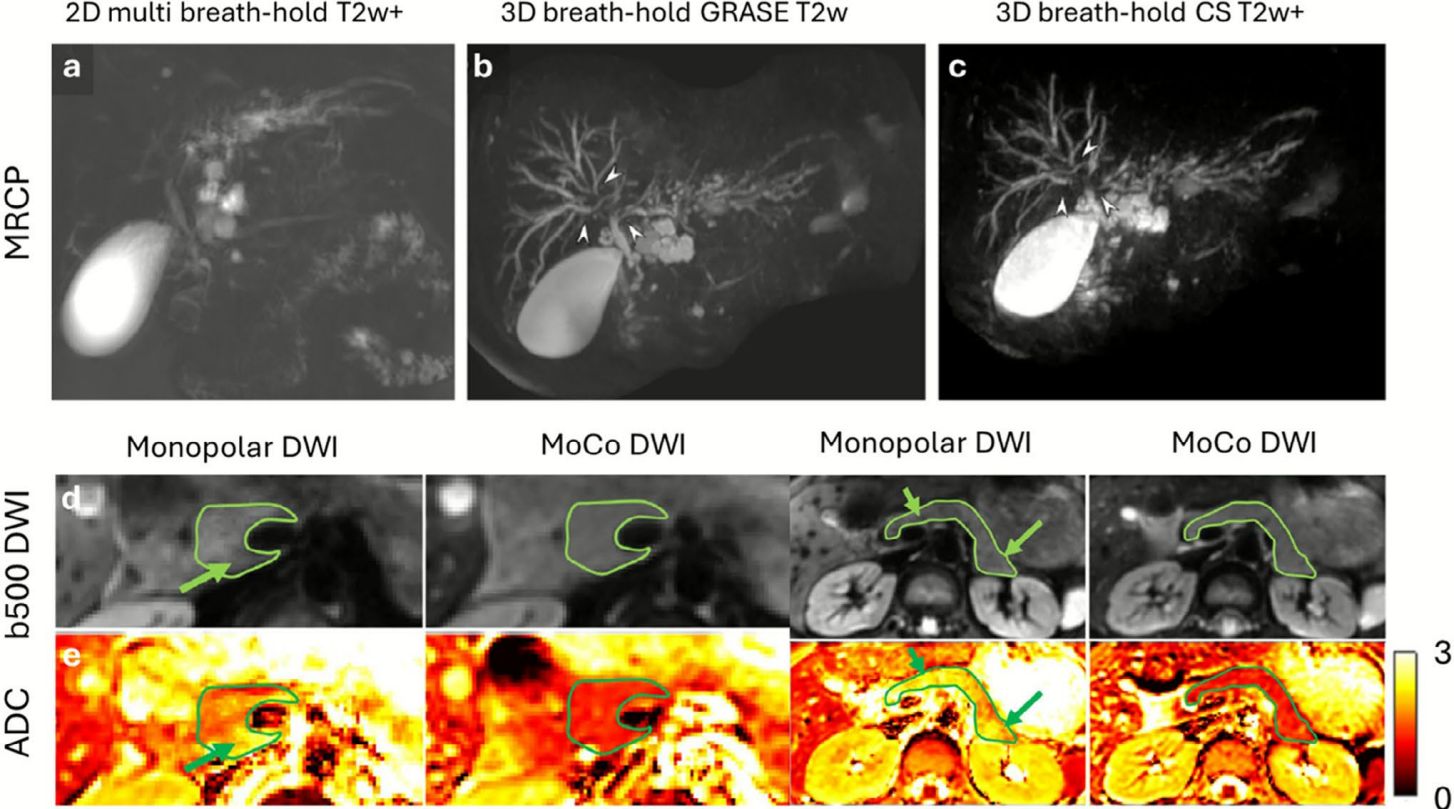
(a–c) Multi breath‐hold T2w + (A) failed likely due to inconsistencies between breath‐holds; CS allowed to accelerate MRCP scans by 10–20×, enabling 3D T2w + single breath‐held MRCP. (d and e) Effects of cardiac pulsation on pancreatic diffusion‐weighted imaging (DWI), in which signal dropout is observed with monopolar diffusion gradients. Homogeneous DWI and ADC signal is observed with the application of velocity compensated diffusion gradients. Adapted from [89, 90].

In heavy T2‐weighted imaging (T2w+), the workhorse of MRCP, the signal for most tissues is effectively nulled except for the long T2 fluids of interest. Consequently, motion‐induced aliasing from tissues outside the ROI (e.g., subcutaneous fat) is reduced. MRCP can be performed in 2D using a single thick slice or with multiple thin slices acquired over multiple breath‐holds. Multi breath‐hold approaches are naturally more sensitive to respiratory drifts and failed or inconsistent breath‐holds (Figure 8c) [87]. Longer 3D MRCP acquisitions under free‐breathing using respiratory navigator gating mitigate these problems with higher SNR and volumetric assessment (Figure 8d), but can have motion artifacts and significantly prolong scan time in patients with irregular breathing patterns [88]. In an effort to further reduce scan time and the need for reliable patient breathing behavior, highly accelerated compressed sensing [13] frameworks have been deployed for MRCP, enabling breath‐held 3D T2w + MRCP [89] (Figure 9a–c).

Another notable challenge of respiratory motion in pancreatic MRI is in MR‐guided radiotherapy: in this application, fast dynamic imaging can be used to track the location of the pancreas. Rather than mitigating motion artifacts, the need is for a low latency framework to capture pancreatic motion in order to minimize accidental chemoradiation delivery to nearby organs like the stomach or duodenum. Accelerated cine bSSFP can be deployed for this purpose to capture dynamic imaging at high temporal resolution, from which pancreatic motion can be derived via template matching and image registration [91]. Recently, advanced techniques like MR‐MOTUS have been developed to estimate patient motion with high spatial–temporal resolution directly from the undersampled k‐space data [92], which could have applications in various MR techniques, including pancreatic MR‐guided radiotherapy.

Pulsation from neighboring vessels can also introduce motion artifacts, potentially creating signal voids that mimic stenosis/strictures (Figure 8e,f) [87]. Cardiac/pulsatile motion has implications in pancreas MRI, as the pulsation can compress the pancreas, causing signal voids in DWI and subsequent positive bias in ADC [90]. This artifact is introduced during the diffusion encoding using monopolar gradients but can be mitigated using velocity compensated diffusion gradients (Figure 9d,e).

### 3.5 Bowel

**FIGURE 10:**
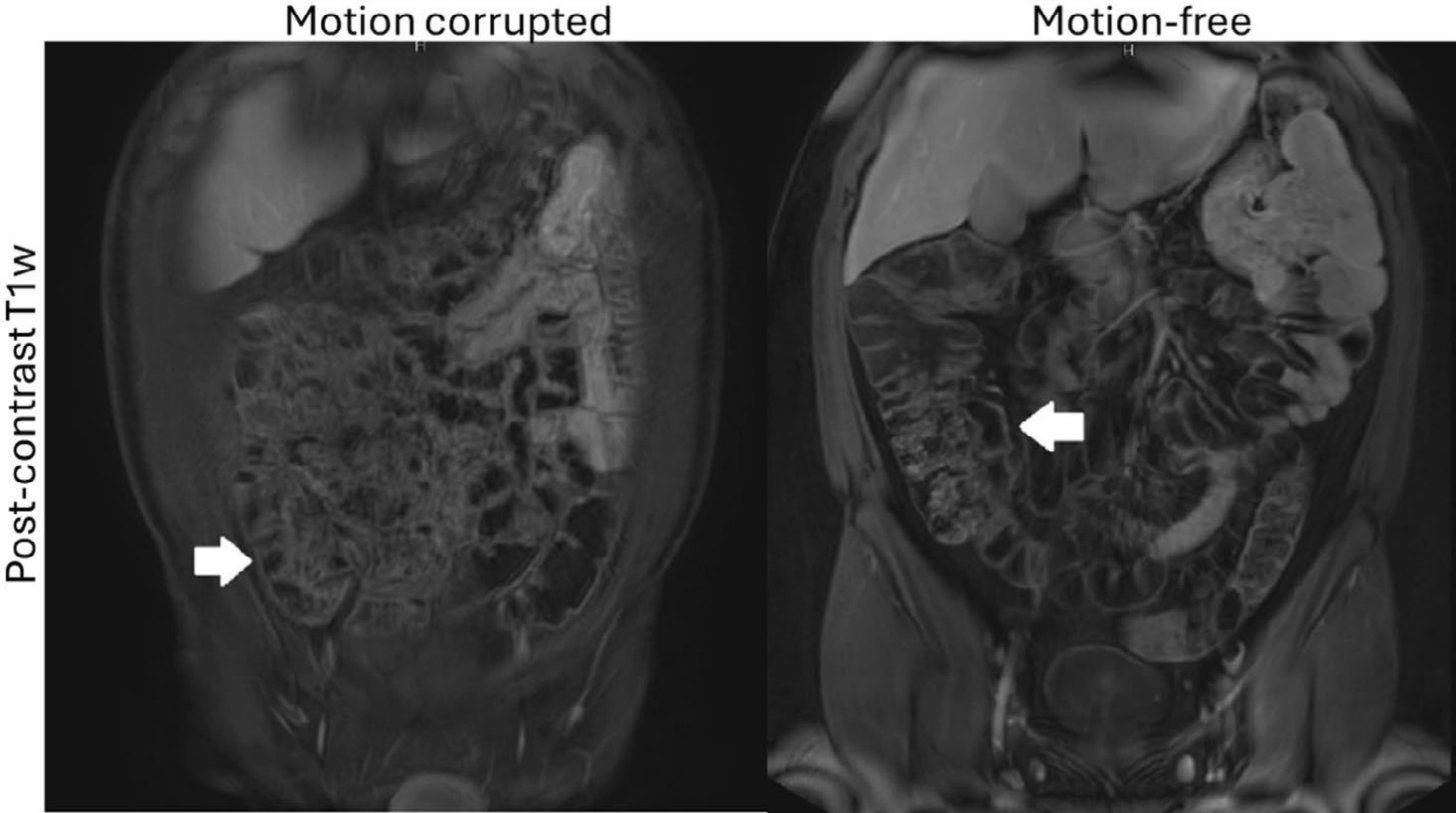
Example post‐contrast T1w imaging of the bowel wall. Peristaltic motion corruption (left) obscures bowel wall visibility, which can be clearly seen if images are taken during a motion‐free (right) state. Adapted from [94].

Small intestine and bowel MRI plays a key role in assessment of Crohn's disease, ulcerative colitis, chronic intestinal pseudo‐obstruction, and other conditions. Fast spin‐echo T2w (with/without fat suppression) plays a key role, supported by (b)SSFP (T2/T1 weighted) and pre‐/post‐contrast fat suppressed T1w imaging, and optionally DWI and cine imaging [93]. Bowel MR is sensitive to respiratory, peristalsis, and bulk motion. Respiratory motion primarily affects the proximal small bowel (i.e., duodenum), having less of an effect in the mid (jejunum) and especially less in the distal small bowel (ileum) [94], where Crohn's primarily occurs. Consequently, breath‐holding has a good success rate in bowel imaging, since even residual respiratory drift is unlikely to produce major artifacts in the ROI. Peristalsis, on the other hand, is a particularly complex type of physiological motion, propagating in a wave‐like pattern along the bowel, mixed with segmental contractions, all without a fixed period. With a motion frequency of ~11 contractions per minute with ~6–7 mm amplitude, peristalsis can produce considerable blurring artifacts on the bowel wall (normal thickness ~3 mm, increasing with inflammation) [6, 95]. These artifacts are exemplified in Figure 10, demonstrating how blurring can significantly obscure the visibility of the bowel wall.

Acquisition of bowel MRI typically employs specific preparation to facilitate the visualization of the bowel wall: fasting to improve bowel distension and reduce peristalsis, biphasic contrast agents e.g., sorbitol for contrast and distention (dark T1w, bright T2w) and potentially gadolinium for T1w; anti‐peristaltic agents e.g., glucagon to freeze bowel motion, breath‐holds to freeze respiratory motion, and prone position to further help with bowel distension and reduce breathing motion (although potentially increasing bulk motion due to discomfort). The logistical overhead associated with the acquisition of bowel MRI underlines the importance for robust protocols with minimal chance of failure. These requirements have challenged the deployment of more advanced MR techniques, although some solutions like free‐breathing DWI [96] (single shot, fat suppressed EPI) and breath‐hold bSSFP tagging [97, 98] have emerged. Of particular interest is cine/real‐time imaging to resolve peristaltic motion, since both the frequency and amplitude of bowel motility are reduced in the presence of inflammation.

**FIGURE 11:**
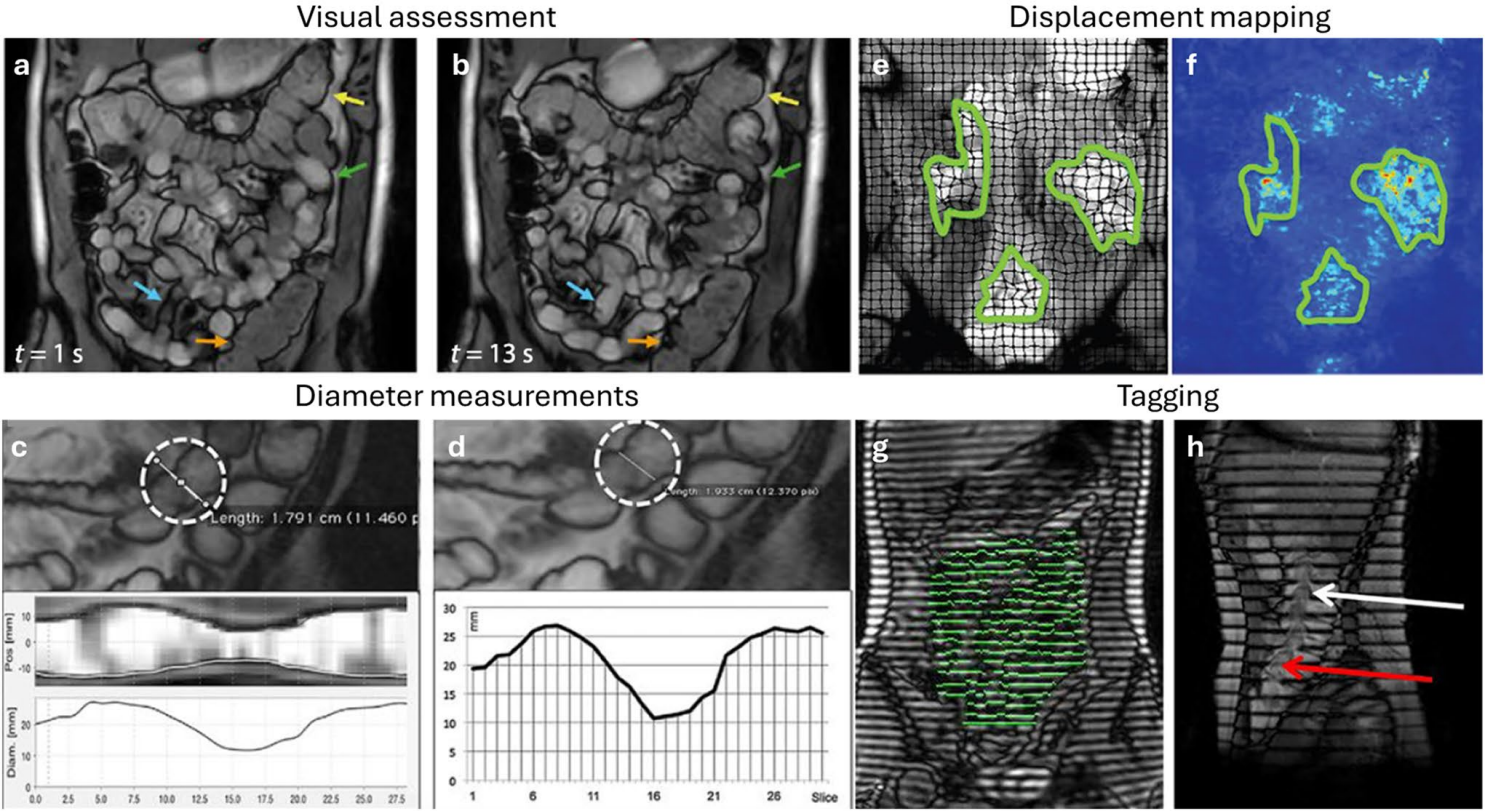
Peristaltic motion in gastrointestinal tract (GI) imaging. (a and b) Dynamic MRI coronal images visualize the GI tract movement over time. Arrows point to different structures: Stomach (red), small bowel (green, blue), and colon (yellow, orange). (c and d) Small bowel diameter measurements can be performed using software assistance (c) or manually with assistance (d). (d–f) Coronal tagging allows for visualization of deformation fields and calculation of displacement. In (g) a coronal GI tagged image is visualized showing deformed taglines in the small bowel n green. In (h) a sagittal image shows movement within the colonic chime, with tag distortion (white arrow) and reduction in tag intensity (red arrow) due to movement. Adapted from [61].

Over the last 10 years, significant advances have been made to visualize and characterize bowel motility [99]. These data are typically acquired during breath‐hold without anti‐peristaltic agents, using bSSFP sequences, enabling sub‐second temporal resolution in 2D or ~1–3 s temporal resolution in 3D. Visualization of the cine dynamic series allows a subjective assessment of motility in various segments of the bowel, which helps diagnose inflammation (Figure 11a,b). Naturally, this method is sensitive to inter‐ and intra‐observer variability. To mitigate this limitation, cross‐sectional diameter measurements of the bowel lumen can be obtained in a manual or semi‐automated fashion, providing quantitative measures of bowel contraction along various segments [10]. However, the process can be time‐consuming and sensitive to respiratory motion or through‐plane motion (Figure 11c,d). More comprehensive motion information can be derived from the cine imaging via image registration, which extracts motion fields for the bowel motion [60]. While bowel motion is useful by itself, efforts have also been made to distill this data into bowel motility score maps using the Jacobian of the motion fields (local compression/expansion) to facilitate characterization (Figure 11e,f) [61]. These techniques have established a negative correlation between bowel motility and symptoms of IBDs like Crohn's in adult [100] and pediatric populations [101], and characterization of intestinal pseudo‐obstruction [102]. Despite currently remaining as an optional sequence, advances in quantitative bowel motility have resulted in robust solutions that are increasingly used clinically and are likely to play a key role in future bowel MRI.

### 3.6 Pediatrics

Pediatric MRI faces many of the same motion challenges as adult imaging (e.g., cardiac, respiratory, peristalsis), but higher heart and respiratory rates necessitate different strategies. Faster heart rates shorten acquisition windows for triggered scans, prolonging overall scan time as a greater total number of heart beats must be included to achieve the same sampling pattern. Pediatric cine imaging also demands higher temporal resolution, further extending scan times. Breath‐holding is often challenging for young children, and rapid respiration reduces the duration of the quiescent end‐expiratory phase. As a result, respiratory navigator scans become less efficient. On the other hand, the relatively smaller amplitude of respiratory motion in pediatric patients may allow for a large enough acceptance window to counteract this issue. As with adults, free‐breathing, or free‐running approaches may help improve efficiency by capturing one or both of the cardiac and respiratory cycles [103, 104], though in practice many pediatric volumetric approaches rely on contrast agents to achieve sufficient SNR.

**FIGURE 12:**
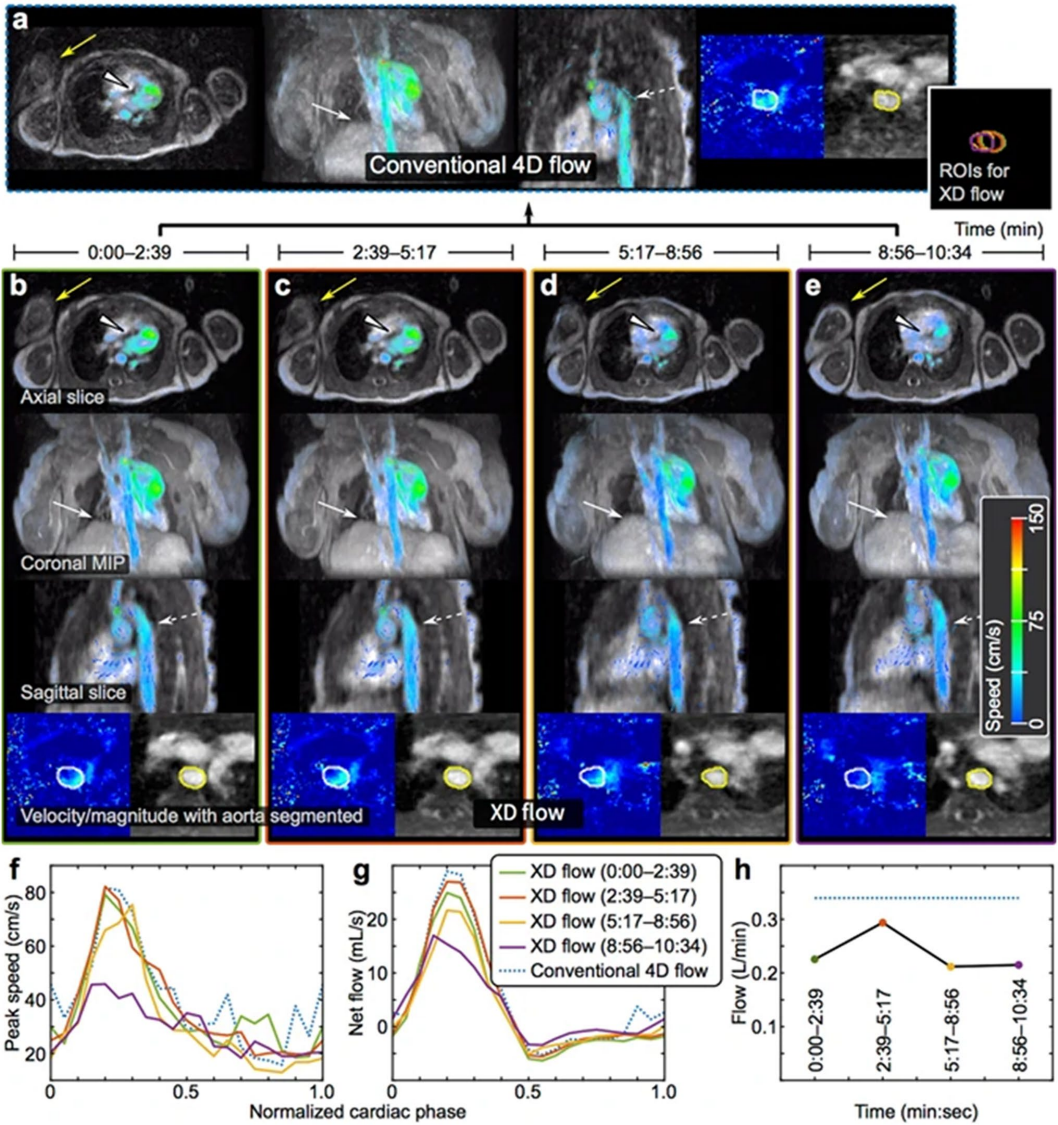
4D flow data in a pediatric patient for anatomical structures and blood flow visualization using a motion robust reconstruction that accounts for respiratory and bulk patient motion by incorporating navigator information and by subdividing the acquisition into sequential time points. (a) Conventional 4D flow data are corrupted by motion. (b–e) Reconstructed subsets of the original data demonstrate motion in the last 2 min of data. (f and h) Flow waveforms and measurements are altered during motion. Adapted from [27].

Perhaps the more significant change when optimizing a protocol for pediatric imaging comes from bulk motion due to patients' behavior (e.g., restlessness, crying, inability to follow breath‐hold instructions) which is typically exacerbated, particularly in younger patients. While effective in reducing voluntary motion, sedation carries risks and is used cautiously, especially in neonates and infants. Motion‐sensitive sequences are often paired with distraction techniques, feed‐and‐wrap protocols, and parental involvement for natural sleep scanning. In the absence of or in combination with these approaches, single‐shot or real‐time imaging may be used to capture the anatomy of interest as fast as possible. This “freezes” bulk motion; however, such approaches may lack the SNR or resolution required. Retrospective correction is another option if motion can be tracked during acquisition. This can be achieved with built‐in navigators or flexible k‐space trajectories that support both real‐time and cine reconstructions [37, 105]. From these data, translational and rotational displacements can be estimated and used to correct k‐space, after which images may be sorted into cardiac or respiratory motion states to generate cine series. Figure 12 demonstrates a motion‐robust approach that incorporates navigators capable of tracking both respiration and bulk patient motion [27].

**FIGURE 13:**
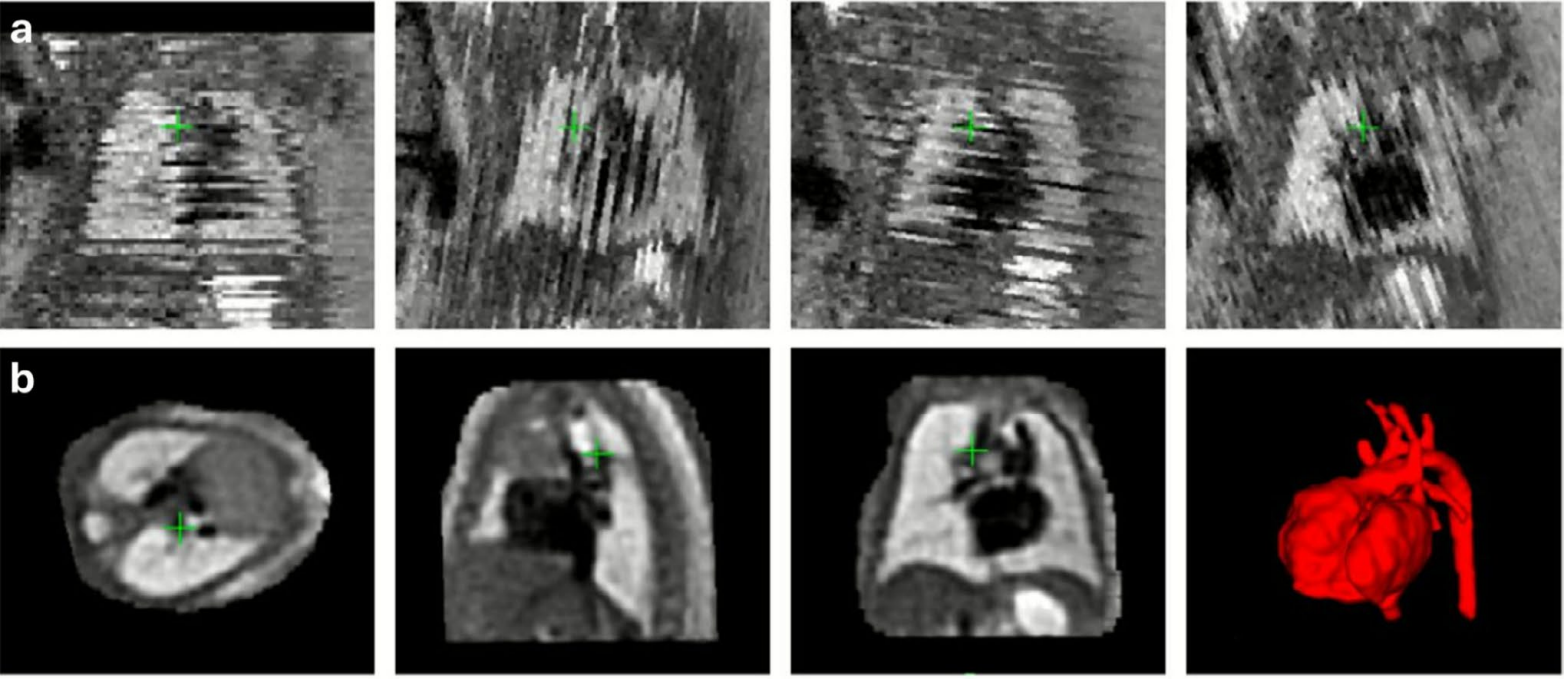
Correction of fetal bulk motion in black‐blood cardiac imaging. (a) Multiple low resolution stacks in different orientations display motion corruption between slices. (b) A slice‐to‐volume (SVR) reconstruction iteratively co‐registers and interpolates misaligned images/stacks to produce an isotropic and motion‐consistent 3D volume. Adapted from [107].

Even with advanced tracking of translational and rotational motion, rapid or complex non‐rigid abdominal movements may exceed the capability of retrospective correction and instead require outlier rejection combined with motion‐robust k‐space trajectories [106]. Bulk motion presents its greatest challenge in fetal MRI, where unpredictable fetal movement is compounded by maternal motion. In particular, the pursuit of volumetric coverage for fetal imaging has largely focused on slice‐to‐volume approaches wherein several stacks of 2D images that can be acquired quickly are co‐registered in 3D space and then interpolated to create a 3D volume that may be static or cardiac motion‐resolved [107, 108]. This approach is particularly successful in correcting both blur within a 2D image as well as spatial misalignment due to motion between the individual slices of the acquired stacks (Figure 13). More recently, the acquisition of 3D volumes for fetal applications has included outlier rejection and correction of translational motion [109].

## 4 Discussion

### 4.1 Strengths and Limitations of Current Motion Mitigation Techniques

Motion mitigation techniques can markedly improve abdominal MRI by reducing blurring and ghosting and salvaging otherwise non‐diagnostic scans. Non‐Cartesian sequences are more robust to motion than Cartesian scans, but this often comes with trade‐offs such as longer acquisition times, reduced spatial resolution, or streak artifacts. Prospective gating and triggering allow motion “freezing” at specific phases of the cardiac or respiratory cycle, but efficiency losses can double scan duration and performance drops with irregular motion. Retrospective strategies, on the other hand, can use all acquired data but require advanced reconstructions with potentially high computational demand and may not yet be routine in clinical workflows. Simple avoidance strategies such as breath‐holds remain valuable but are not feasible for many patients.

### 4.2 Clinical Feasibility and Computational Efficiency

Clinically, the success of motion mitigation depends on ease of integration and reconstruction speed. Vendor‐integrated solutions such as PROPELLER/BLADE or navigator‐based gating are readily available and can be applied with minimal user input, making them the most widely adopted. In contrast, research‐oriented solutions (e.g., compressed‐sensing reconstructions with motion compensation or AI‐driven corrections) can achieve excellent results but may require offline computation or specialized IT infrastructure, limiting their immediate feasibility. Commercial solutions are convenient, regulated, and supported, but costly and sometimes lag behind cutting‐edge methods. Open‐source alternatives are cost‐effective and technically advanced, but require local expertise, are not integrated into vendor workflows, and raise regulatory questions. Bridging this gap by combining the robustness of open‐source methods with the usability of commercial tools will be key to broader adoption.

### 4.3 Impact on Scan Time and Image Reliability

Motion mitigation affects scan time in two ways: some approaches extend acquisition by adding navigators or rejecting data, while others shorten the exam by reducing failed attempts and repeats. In practice, nearly 20% of MRI exams require repeating at least one sequence due to motion, with associated productivity losses estimated at more than $100,000 per scanner annually [1]. By improving first‐pass success rates, motion mitigation often reduces total exam duration despite modest per‐sequence overhead. Importantly, free‐breathing acquisitions are crucial for children, patients with heart failure, or those unable to comply with breath‐holds. Several studies have shown that free‐breathing cardiac cine or abdominal imaging yields diagnostic accuracy comparable to standard breath‐hold protocols, avoiding sedation and improving patient comfort [17, 19, 30, 34, 35, 36, 44, 81, 96, 106]. This reliability translates into more predictable workflows and greater diagnostic confidence.

### 4.4 Barriers to Adoption

Despite the clear advantages of motion mitigation strategies, most evidence still emphasizes technical improvements such as image sharpness rather than clinical or economic outcomes. More studies are needed to quantify its impact on diagnostic accuracy, repeat rates, sedation avoidance, patient satisfaction, and cost savings. Such data would provide a stronger business case for widespread implementation and help identify the most cost‐effective approaches.

At the same time, inconsistent availability remains a major obstacle. Many advanced techniques are restricted to specific scanner models or offered only as optional add‐ons, resulting in uneven adoption between tertiary centers and smaller hospitals. Lack of standardized protocols and variable vendor implementations complicate training and reproducibility, while additional reconstruction steps or delays can reduce acceptance among radiologists and technologists. Greater standardization, education, and vendor support will be essential to make motion mitigation techniques a routine part of clinical workflows.

A further barrier is reliance on external hardware such as external sensors, which adds cost and complexity. More practical are software‐based solutions that exploit motion information already present in the MR signal, including self‐navigation, pilot tone, or AI‐based reconstructions. However, not all types of motion or sensor signals can be recovered solely from software‐based solutions. As vendors increasingly embed motion‐robust sequences and built‐in sensors directly into scanners, motion correction is likely to shift from a specialized option to a standard feature of clinical MRI.

## 5 Emerging Trends and Future Directions

Motion mitigation and correction in MRI is undergoing rapid development, driven by advances in acquisition strategies, sensor technology, and AI. Several emerging trends now point toward broader clinical adoption and improved reliability.

### 5.1 Integration of AI With Motion Correction Techniques

AI‐based reconstructions have already transformed image acceleration and denoising, and their integration with motion correction is a natural next step. AI algorithms can learn motion patterns from raw k‐space or image data and apply corrections that outperform traditional rigid or non‐rigid registration [15, 45, 110]. Models have been applied to reconstruct motion‐resolved images directly from undersampled data, combining motion compensation with compressed sensing in a unified framework [111]. In practice, this could mean free‐breathing cardiac or abdominal scans reconstructed in near real‐time, without the need for external navigators. Moreover, AI offers the potential to separate physiologic motion from pathologic changes, improving diagnostic specificity. For radiologists, this may translate into sharper images with less variability between patients, even in populations traditionally considered challenging, such as children or those with arrhythmias. However, reproducibility, transparency, and regulatory approval remain key hurdles before these techniques become mainstream.

### 5.2 Real‐Time Motion Tracking Using Advanced Sensors

Another promising direction is the use of novel hardware sensors for real‐time motion tracking. Traditional respiratory belts and ECG gating have limitations, particularly in patients with irregular breathing or poor electrode contact. Recent developments include MR‐compatible Doppler ultrasound for cardiac triggering, radar‐based respiratory monitoring, and in‐bore camera systems that track surface motion [20, 38, 112]. These sensors can provide continuous motion information without patient cooperation and can feed directly into prospective correction algorithms. While currently restricted to specialized centers, advances in cost and integration may allow these tools to become part of standard scanner packages. If seamlessly embedded into the scanner workflow, such sensors could help make motion correction invisible to both patients and technologists, while ensuring higher diagnostic confidence for radiologists.

### 5.3 Hybrid Approaches Combining Multiple Correction Methods

Given the multifactorial nature of motion, it is unlikely that any single method will be universally sufficient. Hybrid approaches that combine multiple correction strategies are increasingly explored. Examples include free‐running sequences that retrospectively resolve both cardiac and respiratory motion simultaneously [103, 113, 114], or pipelines that combine prospective motion detection with retrospective image‐domain registration. In addition, motion mitigation strategies are increasingly integrated and paired with reconstruction and analysis methods to guide motion estimation. These multimodal strategies can offer greater robustness, particularly in patients with irregular rhythms or unpredictable motion. Although they add complexity, their value lies in reducing the risk of complete exam failure. For radiologists, the promise of “always diagnostic” imaging, regardless of patient cooperation, represents a powerful incentive for adoption.

### 5.4 Motion Quality Assessment

Automated motion‐quality assessment tools are now emerging to detect and quantify motion artifacts during MRI acquisition, enabling real‐time feedback for rescan decisions. Neural networks can be used for high accuracy in identifying motion‐degraded images without requiring a reference scan, in applications ranging from structural head imaging to pediatric DWI [115, 116]. Deep‐learning‐derived motion severity indices and perceptual similarity functions can provide quantitative scores that could be integrated into MR protocols to enhance reliability and minimize repeat scans. Nevertheless, the identification and validation of motion information and metrics remain a challenge [117].

### 5.5 Toward Fully Automated Motion Correction Pipelines

The long‐term vision is for motion mitigation to become fully automated and integrated into the scanner workflow, requiring no technologist intervention. In such a scenario, motion detection, binning, correction, and reconstruction would occur in real‐time on the scanner's reconstruction hardware, delivering corrected images directly to PACS. Early prototypes already exist, particularly in research contexts [118, 119]. The potential benefits are clear: improved reproducibility across sites, reduced dependence on operator expertise, and a more streamlined exam for patients. For radiologists, the key advantage would be consistent, motion‐robust imaging without additional time penalties or special protocol adjustments. Achieving this vision will require not only technical innovation but also standardization across vendors and robust clinical validation.

### 5.6 Expanding Beyond the Heart: Underexplored Body Applications

While much of the progress in motion correction has been driven by cardiac MRI, other body applications remain underexplored. While the feasibility of lung MRI has increased (with e.g., ultra‐short TE and gas agents), it continues to suffer from respiratory motion. Emerging motion‐robust techniques may help overcome longstanding barriers to broader clinical adoption [120]. Abdominal imaging, particularly of the liver, kidneys, and pancreas, stands to benefit greatly from advanced motion correction, given the combined challenges of respiration, peristalsis, and bulk motion. Small bowel imaging is another area where peristaltic motion remains a significant hurdle. Motion‐aware reconstructions could improve evaluation of inflammatory bowel disease without relying on anti‐peristaltic agents. Whole‐body and pediatric imaging also remain areas where robust, patient‐independent motion correction would provide substantial value. Pelvic MRI adds further complexity, particularly in female patients. In addition to respiration and bowel peristalsis, uterine and ovarian motion can affect image quality, while lengthy protocols for conditions such as endometriosis or fertility assessment increase vulnerability to bulk motion. Free‐breathing, motion‐corrected protocols could improve diagnostic accuracy in gynecologic oncology, adenomyosis, and infertility assessments, while helping to standardize image quality across centers. Despite its clinical relevance, motion mitigation techniques in these areas remain limited.

Motion mitigation and correction remains one of the central challenges in body and cardiac MRI. Despite progress, current methods still face limitations in efficiency, integration, and availability. Many techniques are confined to research settings, integration across vendors is inconsistent, and robust evidence on cost‐effectiveness and diagnostic impact is still lacking. Addressing these gaps represents a clear opportunity for the field, both in terms of technology development and clinical validation. Extending techniques beyond the heart to abdominal, pelvic, pediatric, and whole‐body imaging is particularly important, as these areas remain underexplored but highly relevant for clinical care.

For clinical practice, reliable motion‐robust imaging could reduce repeat scans, sedation, and variability across sites, ultimately improving diagnostic confidence and patient experience. For research, the development of standardized protocols, open‐source tools, and multi‐center studies will be essential to translate technical advances into clinical benefit.

The next generation of motion mitigation solutions, from AI‐driven reconstructions and advanced sensors to hybrid strategies and automated pipelines, suggests a future where motion correction is seamlessly integrated and becomes a core feature of MRI.

## Funding

Gastao Lima da Cruz funding from NIH R01 HL153034. Christopher W. Roy funding from Swiss National Science Foundation Grant PZ00P3_202140. Thomas Küstner funding from German Research Foundation under Germany's Excellence Strategy—EXC 2064/1—Project number 390727645.
